# Complete Mitogenomes of Two *Aragoa* Species and Phylogeny of Plantagineae (Plantaginaceae, Lamiales) Using Mitochondrial Genes and the Nuclear Ribosomal RNA Repeat

**DOI:** 10.3390/plants10122673

**Published:** 2021-12-05

**Authors:** Jeffrey P. Mower, Lilly Hanley, Kirsten Wolff, Natalia Pabón-Mora, Favio González

**Affiliations:** 1 Center for Plant Science Innovation, University of Nebraska, Lincoln, NE 68588, USA; noemail@noemail.edu; 2 Department of Agronomy and Horticulture, University of Nebraska, Lincoln, NE 68583, USA; 3 School of Natural and Environmental Sciences, Newcastle University, Newcastle Upon Tyne NE1 7RU, UK; kirsten.wolff@newcastle.ac.uk; 4 Instituto de Biología, Universidad de Antioquia, Medellín 050010, Colombia; lucia.pabon@udea.edu.co; 5 Sede Bogotá, Facultad de Ciencias, Instituto de Ciencias Naturales, Universidad Nacional de Colombia, Bogotá 111321, Colombia; fagonzalezg@unal.edu.co

**Keywords:** *Aragoa*, mitochondrial genome, nuclear rRNA, phylogenetics, Plantagineae

## Abstract

*Aragoa*, comprising 19 high-altitude North Andean species, is one of three genera in the Plantagineae (Plantaginaceae, Lamiales), along with *Littorella* and *Plantago*. Based primarily on plastid data and nuclear ITS, *Aragoa* is sister to a clade of *Littorella* + *Plantago*, but Plantagineae relationships have yet to be assessed using multigene datasets from the nuclear and mitochondrial genomes. Here, complete mitogenomes were assembled for two species of *Aragoa* (*A. abietina* and *A. cleefii*). The mitogenomes of both species have a typical suite of genes for 34 proteins, 17 tRNAs, and three rRNAs. The *A. abietina* mitogenome assembled into a simple circular map, with no large repeats capable of producing alternative isoforms. The *A. cleefii* mitogenomic map was more complex, involving two circular maps bridged by a substoichiometric linear fragment. Phylogenetics of three mitochondrial genes or the nuclear rRNA repeat placed *Aragoa* as sister to *Littorella* + *Plantago*, consistent with previous studies. However, *P. nubicola*, the sole representative of subg. *Bougueria*, was nested within subg. *Psyllium* based on the mitochondrial and nuclear data, conflicting with plastid-based analyses. Phylogenetics of the nuclear rRNA repeat provided better resolution overall, whereas relationships from mitochondrial data were hindered by extensive substitution rate variation among lineages.

## 1. Introduction

*Aragoa* is a genus of 19 woody shrubs that reside at high altitudes in the páramos of the Northern Andes of Colombia and Venezuela [1,2,3]. Based on analyses of nuclear ITS, various plastid genes, or the entire plastid genome [3,4,5,6,7,8], *Aragoa* was shown to be sister to *Plantago* (~250 species) and *Littorella* (three species), which altogether make up the Plantagineae [6]. Recently, whole plastome data [8,9] and combined 5-loci analyses from all three plant genomes [10] have shown promise for resolving relationships among Plantagineae and within *Plantago*. However, sampling of nuclear and mitochondrial loci has been limited to nuclear ITS [3,4,5,6,10] and single mitochondrial genes [10,11]. Thus, it is currently unclear whether the plastome results are congruent with the phylogenetic signal in the mitochondrial and nuclear genomes.

To begin to assess the mitogenomic signal, we sequenced and assembled complete mitogenomes for two species of *Aragoa* (*A. abietina* and *A. cleefii*), which represent distinct groups within the genus [1,2]. We augmented these genomic data with published sequences for three mitochondrial genes (*atp1*, *cox1*, and *rrnS*) that have been widely sequenced from previous Plantaginaceae studies [11,12], and by extracting these mitochondrial genes from Illumina assemblies of 10 additional Plantaginaceae species. In parallel, we performed an analysis of the nuclear ribosomal RNA (rRNA) cluster, which includes genes for 18S, 5.8S, and 26S rRNAs separated by ITS1 and ITS2 regions. This dataset combined available data from GenBank with extracted nuclear rRNA sequences present in previous assemblies used to capture the plastid genome [8] and from the assemblies performed in this study. Using these data, we describe the complete mitogenomes of two *Aragoa* species and compare the phylogenetic signal for Plantagineae relationships using mitochondrial and nuclear loci with previous plastid phylogenomic results.

## 2. Results

### 2.1. Aragoa Mitogenomic Content and Structure

**Figure 1:**
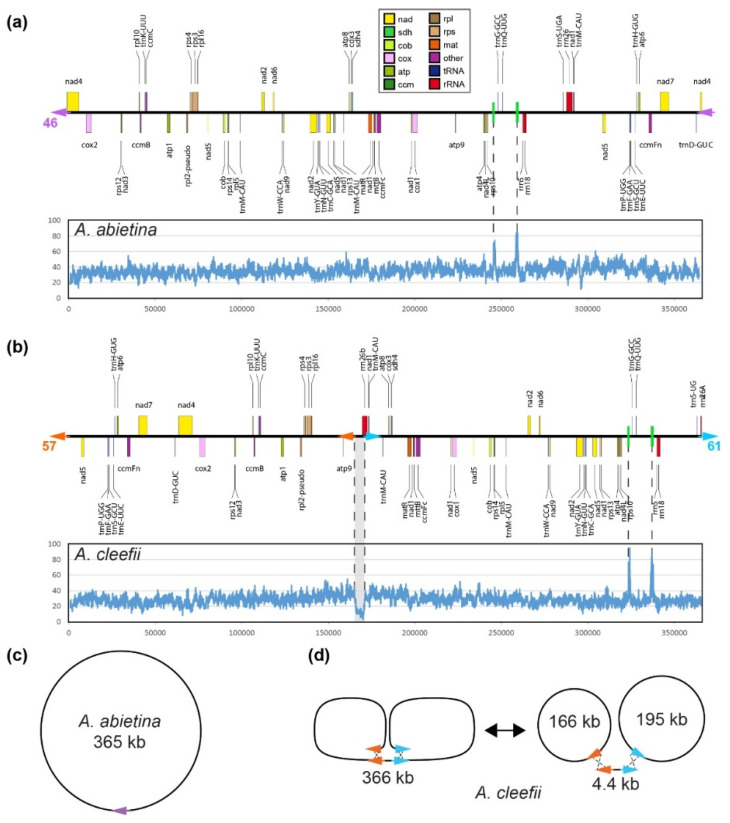
Linear gene map and depth of sequencing coverage for *Aragoa* mitogenomes. (**a**) *Aragoa abietina.* (**b**) *Aragoa cleefii*. Genes are color coded according to their functional group as shown in the key at the top. All MIPTs >500 bp are shown in green. Positions of atypically high or low coverage are indicated with dotted lines connecting the coverage plot and gene map. Arrowheads indicate structural connections supporting a circularized assembly, and the number of read pairs supporting these connections are indicated. (**c**) Inferred circularized assembly for the *A. abietina* mitogenome. (**d**) Inferred assembly structures for *A. cleefii*, including the putative interconversion of a linearized form and a set of circular and linear forms. Note that these are assembled structures only and may not represent in vivo forms.

The mitochondrial genomes of *A. abietina* (365 kb) and *A. cleefii* (366 kb) were assembled from an Illumina paired-end library with a large-insert size (~800 bp). In terms of annotated content, both mitogenomes contain the same set of intact and putatively functional genes encoding 34 proteins, 17 transfer RNAs (tRNAs), and three rRNAs (Figure 1a,b). Repeat content includes the same set of 10 repeats in both species (minimum 100 bp in length and >90% sequence identity), the largest of which is only 483 bp in length. Both mitogenomes also contain the same 15 segments of mitochondrial DNA originating from plastid (MIPTs, minimum 100 bp in length and 80% sequence identity), of which two are >1 kb in size at 1.7 kb and 1.3 kb (Figure 1a,b).

Read-pairs were mapped to each genome to evaluate depth of sequencing coverage and also to assess the positional consistency of each read pair, which should map in a head-to-head orientation at an approximate distance of the library insert size (800 bp ± 50%). For *A. abietina*, depth of coverage was consistent across the mitogenome (mean 35.0×, median 34.6×), except for two coverage spikes at positions 246 and 259 kb in the assembly, corresponding to the two largest MIPTs (Figure 1a). Nearly all read pairs mapped in a consistent manner, except that 46 read pairs mapped at the two ends of the scaffold, providing support for a circular mapping assembly (Figure 1c).

For the *A. cleefii* mitogenome, coverage depth was also generally consistent (mean 28.4×, median 28.1×), except for two coverage spikes at the two largest MIPTs at positions 324 and 337 kb, and for a substantial dip in coverage (mean and median = 10.9×) at a 4.4 kb region at position 165,852–170,296 (Figure 1b). Read pairs also mapped consistently to the *A. cleefii* mitogenome assembly, except for 57 read pairs that mapped to the left end of the scaffold and the left flank of the low-coverage region, and another 71 read pairs that mapped to the right end of the scaffold and the right flank of the low-coverage region. Thus, the coverage analysis and read-pair consistency mapping support a complex assembly involving two circular maps of 195 and 166 kb and a substoichiometric linear segment of 4.4 kb (Figure 1d). Notably, the 195 kb circular map reconstructs a contiguous *rrn26* gene that was split into two distally spaced pieces in the linear arrangement shown in Figure 1b.

No other inconsistently mapping clusters of read pairs (minimum support of five clustered read pairs in a 1 kb window) were detected in either *Aragoa* mitogenome, indicating that no other alternative isoforms exist. This finding is consistent with the fact that neither genome has a large repeat (>1 kb) that might promote high-frequency repeat-mediated rearrangement.

### 2.2. Plantagineae Phylogeny Based on Three Mitochondrial Genes

**Figure 2:**
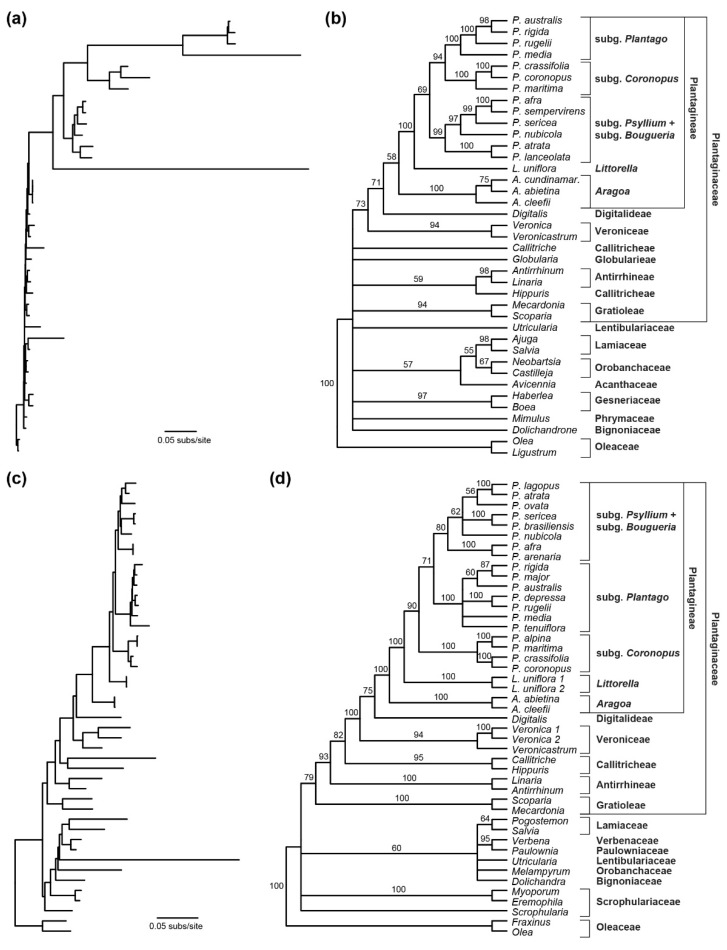
Phylogenetic relationships among Plantaginaceae tribes and Plantagineae genera. (**a**) Phylogram and (**b**) cladogram based on three concatenated mitochondrial genes. (**c**) Phylogram and (**d**) cladogram based on the nuclear rRNA cluster. Branches with <50% bootstrap support were collapsed in the cladograms.

The phylogram from a phylogenetic analysis of three mitochondrial genes (*atp1*, *cox1*, and *rrn18*) indicates substantial rate variation among Plantagineae taxa (Figure 2a), with particularly high levels of sequence divergence for *Littorella* and *Plantago*, as described previously [11,13]. The cladogram (Figure 2b) indicates that Plantagineae is weakly supported as monophyletic (BS = 58%) and sister to Digitalideae (represented by *Digitalis*) with moderate support (71%). Within Plantagineae, *Aragoa* is monophyletic (100%) and sister to a clade comprising *Littorella* and *Plantago* (100%), and *Littorella* is moderately supported as sister to a monophyletic *Plantago* (69%). Among *Plantago* subgenera, the monotypic subg. *Bougueria* (represented by *P. nubicola*) is nested within subg. *Psyllium* with strong support (97%), and a sister relationship between subgenera *Coronopus* and *Plantago* is strongly supported (94%).

Single-gene phylogenetic analyses of each mitochondrial gene were also performed (Figure S1). All three single-gene trees provide good support for the monophyly of subg. *Plantago* (BS = 100%, 99%, and 100% for *atp1*, *cox1*, and *rrn18*, respectively) and for the clade comprising *Littorella* + *Plantago* (79%, 99%, and 95%). Most other relationships are somewhat inconsistent among trees and received generally poor bootstrap support, suggesting limited phylogenetic signal in the single-gene datasets.

### 2.3. Plantagineae Phylogeny Based on the Nuclear rRNA Repeat

A phylogram, based on the nuclear rRNA repeat cluster, indicates that this locus exhibits much less rate variation among species, with increased divergence limited to *Utricularia* (Figure 2c). The cladogram indicates that the bootstrap support is generally more robust for this nuclear dataset (Figure 2d). Consistent with the mitochondrial results, Plantagineae is shown to be monophyletic (100%) and sister to Digitalideae (75%), and *Aragoa* and *Littorella* are successive sisters (100%, 100%) to a monophyletic *Plantago* (90%). Moreover, *P. nubicola* is again found to be nested within subg. *Psyllium*, although with limited support (62%). In contrast to the mitochondrial results, the subg. *Bougueria* + subg. *Psyllium* clade associates with subg. *Plantago* (71%), placing subg. *Coronopus* as sister to the other *Plantago* subgenera with moderate support.

## 3. Discussion

Organellar genomes often assemble into circular maps. However, the predominant mitogenomic structure for many eukaryotes (particularly plants and fungi) is not a circular chromosome but instead a collection of linear molecules of variable size and more complex multibranched linear forms [14,15]. In plants, multiple isoform maps are often inferred due to recombination at large repeats that promote genomic rearrangements [16]. Here, the *A. abietina* mitogenome also mapped as a circular molecule (Figure 1b,d). However, the absence of large repeats (>1 kb) indicates that this mitogenome map has no major alternative isoforms, which was corroborated by the absence of inconsistently mapping read pairs that would indicate a major alternative arrangement. The lack of any large, recombinogenic repeats is uncommon for a plant mitogenome, although some other plants also lack large mitochondrial repeats, such as *Brassica hirta* [17], *Batis maritima* [18], *Ajuga reptans* [19], and 13 of 72 angiosperms evaluated in a broader study [20].

The assembly map for the *A. cleefii* mitogenome was more atypical. The genome assembled into a 366 kb linear scaffold; however, a 4.4 kb internal segment was clearly substoichiometric (by about a factor of 3) relative to the rest of the assembly (Figure 1b), indicating that this assembled form does not accurately represent the in vivo stoichiometry of the mitogenome. Moreover, no read-pair clusters were found to span the two ends of the 366 kb linear scaffold, arguing against a single circularized map. Instead, substantial read pairs supported an assembly involving two independent circular maps that are infrequently interconnected by the substoichiometric 4.4 kb linear segment (Figure 1d). In some sense, the linearization of the *A. cleefii* circular maps by a small linear segment is reminiscent of linear plasmids that linearize the maize CMS-S mitogenome [21,22]. However, the 4.4 kb linear segment in *A. cleefii* does not have any terminal inverted repeats or evidence of genes (such as DNA or RNA polymerase) typical of a mitochondrial plasmid [22,23], arguing against the possibility that this 4.4 kb segment is a mitochondrial plasmid. Similar to *A. cleefii*, the mitogenome maps for cytoplasmic male-sterile lines of carrot and beet could not be completely circularized, with some fraction of the genome remaining linearized [24,25], suggesting that noncircular maps may be more common among plants than currently recognized. The in vivo structure of the *A. cleefii* mitogenome requires further investigation.

The nuclear and mitochondrial datasets examined here (Figure 2) represent the largest sets of nuclear and mitochondrial genes for Plantagineae phylogenetics. In general, the results of these analyses are consistent in several respects with previous analyses based on whole plastid genomes [8], single plastid genes, and/or the nuclear ITS [3,4,5,10], and a combined analysis of five mitochondrial, plastid, and nuclear loci [10]. Collectively, these studies demonstrate that Plantagineae is monophyletic, with *Aragoa* and *Littorella* as successive sisters to a monophyletic *Plantago*. The consistency among studies, and particularly among the three plant genomes, indicates that these conclusions are robust. Moreover, the inferred relationships are unlikely to be influenced by the minor differences in taxon sampling among datasets. The results of this study and the previous plastome-based study [8] also suggest that Plantagineae may be most closely related to Digitalideae, although not all Plantaginaceae tribes were sampled in either study. Resolution of relationships among Plantaginaceae tribes will require more extensive sampling of all tribes from all three plant genomes.

Within *Plantago*, relationships inferred from the mitochondrial and nuclear analyses from this study and the predominantly plastid-based analyses of previous studies were less consistent. For example, plastome data strongly supports a sister-group relationship between subg. *Bougueria* and subg. *Psyllium* [4,8], whereas subg. *Bougueria* is nested within subg. *Psyllium* based on the mitochondrial and nuclear datasets examined here (Figure 2). In addition, subg. *Coronopus* is sister to subg. *Plantago* in the mitochondrial and plastid analyses but sister to the remainder of *Plantago* based on the nuclear rRNA data. Phylogenetic incongruence among genomes can have a variety of causes, ranging from technical (e.g., limited taxon or locus sampling) to biological (e.g., introgression, incomplete lineage sorting, reticulation). Here, it should be noted that previous whole plastome studies [8,9] analyzed a much larger dataset relative to the mitochondrial and nuclear datasets examined here; additional sampling of nuclear and mitochondrial loci is likely to improve support values for these analyses. 

Finally, the extreme rate variation in the mitochondrial dataset suggests that the mitochondrial results should be interpreted with caution. In one sense, the higher mitochondrial substitution rates in *Littorella*, subg. *Coronopus*, and subg. *Plantago* may be beneficial in producing an abundance of variable characters. However, the reliability of the inferred relationships may be negatively affected by issues of long-branch attraction. Given the incongruence among genomes for *Plantago* relationships, and the huge variation in mitochondrial substitution rates among taxa, we urge caution in combining datasets derived from different genomes for the study of *Plantago* phylogenetics.

## 4. Materials and Methods

### 4.1. Generation of Aragoa Mitochondrial Genomes

Tissue was collected in Colombia and silica-dried for two species of *Aragoa*: *A. abietina* [voucher *Natalia Pabón-Mora & Favio González 288* (COL, HUA)] and *A. cleefii* [voucher *Favio González 4614* (COL, HUA)]. DNA was extracted by use of a CTAB-based protocol [26] and then sequenced by BGI (Shenzhen, China) on an Illumina HiSeq 2500 machine to generate 6.3 Gb (for *A. abietina*) or 7.0 Gb (for *A. cleefii*) of 2 × 125 paired-end sequence data from an 800 bp library (Table S1). Illumina sequence data for both species were assembled with Velvet v1.2.10 [27] and SPAdes v3.11.1 [28], using methods described previously [29]. For each species, mitochondrial contigs were identified using blastn v2.2.31 (with default settings) with a set of mitochondrial reference genes found in angiosperms [30] as queries. A consensus mitogenome sequence was generated by manual alignment of overlapping mitochondrial contigs from the Velvet and SPAdes assemblies.

For each *Aragoa* mitogenome, protein and rRNA genes were annotated by identifying their position using blastn with the mitochondrial reference genes as queries. When necessary, start and stop codon positions were manually adjusted if their position shifted in *Aragoa* relative to the reference genes. Genes for tRNAs were identified using tRNAscan-SE [31] as implemented in the GeSeq web portal [32].

Depth of coverage was calculated as described previously [33]. Briefly, Illumina read-pairs were mapped to the complete genome with blastn v2.2.31, requiring a minimum match of 90% length and 90% sequence identity. Read-pairs were classified as “consistent” if they mapped in a head-to-head orientation at a distance consistent with the library insert size (800 bp ± 50%). Read-pairs that did not map consistently were classified as “inconsistent” and were binned in 1 kb windows. Clusters of inconsistent read pairs were identified when a minimum of 5 read pairs mapped to the same pair of 1 kb windows.

### 4.2. Generation of Mitochondrial Gene Sequences

DNA from *Littorella uniflora*, *Plantago afra*, and *Plantago nubicola* was extracted from fresh or silica-dried material and Illumina sequenced using the same procedures described above for *Aragoa* (Table S1). Illumina or Ion Torrent genome sequencing data were obtained from the NCBI SRA for seven additional Plantagineae species (Table S1). Sequence data for these ten species were assembled with Velvet v1.2.10 and SPAdes v3.11.1 as described above for *Aragoa*. For each species, contigs containing the mitochondrial genes *atp1*, *cox1*, and *rrn18* were identified using blastn version 2.2.31 (with default settings), and the sequences of these genes were directly extracted from the contigs. For *A. cundinamarcensis*, genomic DNA was obtained from the Royal Botanic Gardens (Kew, U.K.) DNA Bank (accession ID 11177), and the *atp1* gene was amplified using primers and PCR conditions as described previously [12]; the mitochondrial *rrn18* and nuclear rRNA loci were not amplified from this DNA. These newly generated mitochondrial gene sequences were deposited in GenBank, and sequences from additional species were obtained from GenBank (Table S2).

### 4.3. Generation of Nuclear Ribosomal RNA Sequences

During the sequencing and assembly of Plantagineae plastid genomes [8] and/or the draft mitogenomes for this study (Table S1), the nuclear rRNA repeat cluster (including 18S, ITS1, 5.8S, ITS2, 26S) was usually recovered in one or a few contigs. These contigs containing nuclear rRNA sequences were identified using blastn version 2.2.31 (with default settings), and then they were manually aligned based on sequence overlap to build a consensus sequence of the entire nuclear rRNA cluster. The 18S, 5.8S, and 26S rRNA genes were annotated based on comparison to the annotated nuclear rRNA clusters from *Eremophila crassifolia* and *Paulownia coreana* (GenBank accessions MN411425 and KP718623, respectively). The newly generated nuclear rRNA sequences were deposited in GenBank and supplemented with additional sequences available in GenBank (Table S3).

### 4.4. Phylogenetic Analyses

Sequences of mitochondrial *atp1*, *cox1*, and 18S rRNA genes (Table S2) and the nuclear rRNA repeat cluster (Table S3) were aligned using MUSCLE version 3.8.31 [34]. Ambiguously aligned regions in each dataset were trimmed using Gblocks version 0.91b [35] with relaxed parameter settings (−t = d − b1 = half − b2 = half − b3 = 8 − b4 = 5 − b5 = half). A trimmed, concatenated 3-gene mitochondrial dataset was also created using Gblocks in batch mode (−a = y) with the same relaxed parameter settings. Phylogenetic analyses of each trimmed dataset were performed using RAxML version 8.2.4 [36] with a GTR+G+I model (general time-reversible nucleotide model with gamma-distributed rate variation and a proportion of invariant sites) and 1000 rapid bootstrap replicates.

## Data Availability

All newly generated data were deposited in GenBank under accessions OK514181, OK514182, OK523398–OK523436, OK559378–OK559395, and OK959863–OK959867. See Tables S2 and S3 for more details.

## Supplementary Materials

The following are available online at https://www.mdpi.com/article/10.3390/plants10122673/s1, Supplementary File S1: File includes Figure S1, Tables S1–S3.
