# Supplementary material for: Complete Mitogenomes of Two Aragoa Species and Phylogeny of Plantagineae (Plantaginaceae, Lamiales) Using Mitochondrial Genes and the Nuclear Ribosomal RNA Repeat
Source: Plants (Basel). 2021 Dec 5;10(12):2673. doi: 10.3390/plants10122673 (PMC8707427; doi:10.3390/plants10122673)
Supplement: Supplementary file 1 [file plants-10-02673-s001.zip › plants-1493496-supplementary.pdf]

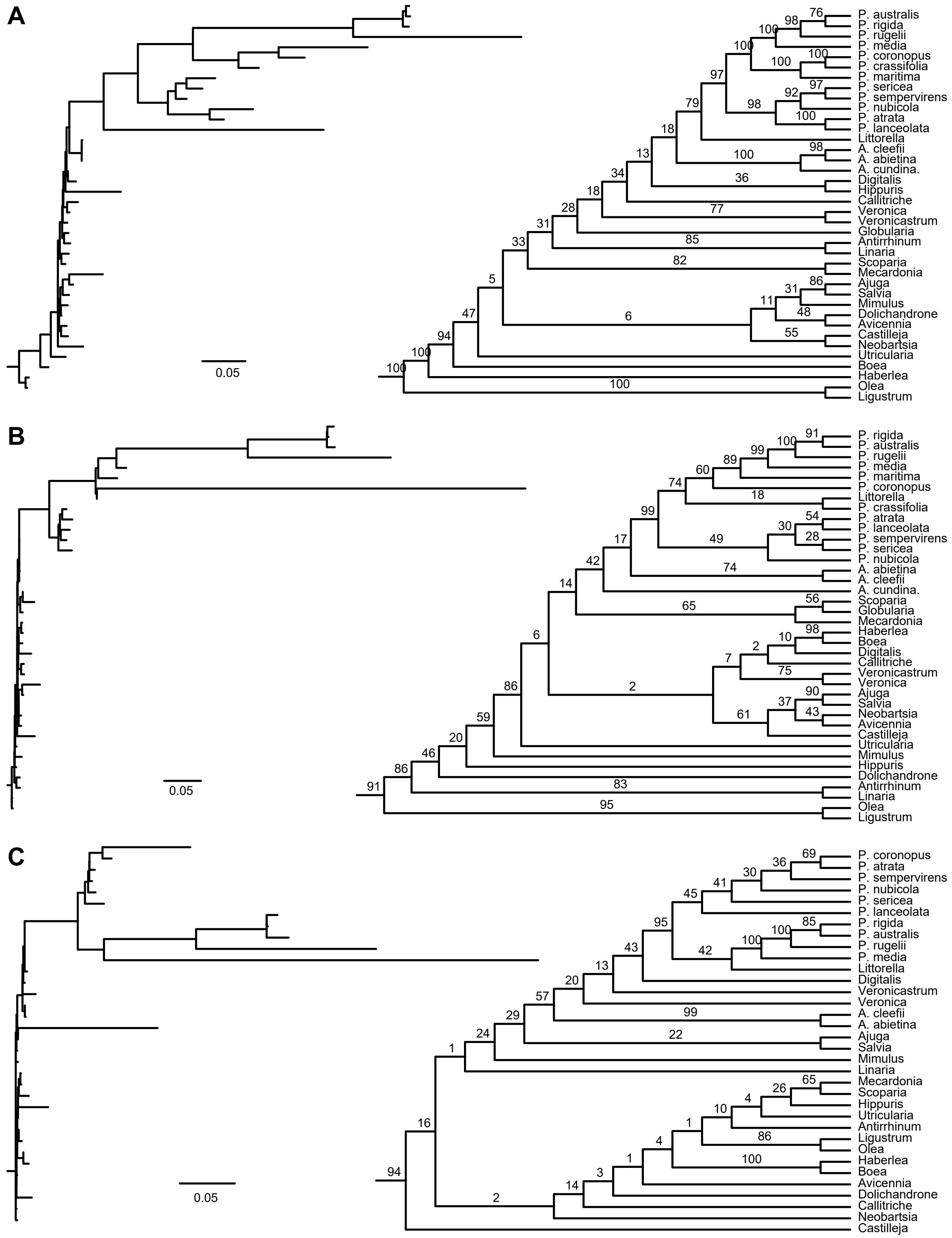

Figure S1. Maximum likelihood phylograms (left) and cladograms (right) from individual analysis of mitochondrial A) *atp1*, B) *cox1*, and C) *rrn18*. Asterid outgroups (*Daucus*, *Helianthus*, *Panax*) not shown.

Table S1. Source of plant materials and sequencing information

Species	Source	Sequence information
Antirrhinum majus	read data from NCBI SRA (ERR2124222)	Illumina Hiseq 2x90 (14.5 Gb)
Aragoa abietina	dried tissue from Colombia [voucher N Pabón-Mora & F González 288 (COL, HUA)]	Illumina HiSeq 2x125 (6.3 Gb)
Aragoa cleefii	dried tissue from Colombia [voucher F González 4614 (COL, HUA)]	Illumina HiSeq 2x125 (7.0 Gb)
Aragoa cundinamarcensis	DNA from Kew DNA Bank (ID 11177)	Sanger sequenced PCR amplicons
Callitriche palustris	read data from NCBI SRA (SRR14844933)	Illumina HiSeq 2x150 (3.5 Gb)
Hippuris vulgaris	read data from NCBI SRA (SRR12879273)	Illumina BGISeq 2x150 (3.1 Gb)
Linaria vulgaris	read data from NCBI SRA (SRR2156275)	Ion Torrent avg 268 bp (1.0 Gb)
Littorella uniflora	live plant from Štěpán Husák (Czech Acad. Sciences)	Illumina HiSeq 2x100 (9.4 Gb)
Mecardonia procumbens	read data from NCBI SRA (SRR7121950)	Illumina BGISeq 2x100 (74 Gb)
Plantago afra	seeds from Dijon Botanical Garden	Illumina HiSeq 2x150 (2.2 Gb)
Plantago nubicola	dried tissue from Stephan Beck (Herbario Nac. Bolivia) [SG Beck 34557 (LPB)]	Illumina HiSeq 2x125 (6.4 Gb)
Scoparia dulcis	read data from NCBI SRA (SRR7121581)	Illumina BGISeq 2x100 (60 Gb)
Veronicastrum axillare	read data from NCBI SRA (SRR13607868)	Illumina NovaSeq 2x149 (3.5 Gb)

Table S2. Accession numbers for mitochondrial genes

Species	*atp1*	*cox1*	*rrn18*
**Plantaginaceae**			
Antirrhinum majus	**OK523427**	**OK559378**	**OK559387**
Aragoa abietina	**OK514181**	**OK514181**	**OK514181**
Aragoa cleefii	**OK514182**	**OK514182**	**OK514182**
Aragoa cundinamarcensis	**OK523428**	EU069509	–
Callitriche palustris	**OK523429**	**OK559379**	**OK559388**
Digitalis purpurea	AY741841	AJ223415	AF193999
Globularia punctata	AY741842	EU156494	–
Hippuris vulgaris	**OK523430**	**OK559380**	**OK559389**
Linaria vulgaris	**OK523431**	**OK559381**	**OK559390**
Littorella uniflora	**OK523432**	**OK559382**	**OK559391**
Mecardonia procumbens	**OK523433**	**OK559383**	**OK559392**
Plantago afra	**OK959863**	**OK959864**	**OK959865**
Plantago atrata	AY818936	EU069536	AJ389618
Plantago australis	AY741847	AJ389608	AY818949
Plantago coronopus	AY741843	AJ389609	AJ389617
Plantago crassifolia	AY741844	EU069516	**OK959866**
Plantago lanceolata	AY818937	AJ389611	AJ389619
Plantago maritima	HQ593805	EU069522	**OK959867**
Plantago media	AY818938	AJ389605	AJ389614
Plantago nubicola	**OK523434**	**OK559384**	**OK559393**
Plantago rigida	AY741848	AJ389607	AJ389616
Plantago rugelii	AY818939	AJ389606	AJ389615
Plantago sempervirens	AY818940	AJ389612	AJ389620
Plantago sericea	AY818941	AJ389613	AJ389621
Scoparia dulcis	**OK523435**	**OK559385**	**OK559394**
Veronica spp.	AY818943	AJ223427	AY818950
Veronicastrum axillare	**OK523436**	**OK559386**	**OK559395**
**Other Lamiales**			
Ajuga reptans	KF709392	KF709392	KF709392
Avicennia marina	CM032784	CM032784	CM032784
Boea hygrometrica	JN107812	JN107812	JN107812
Castilleja paramensis	KT959112	KT959112	KT959112
Dolichandrone cauda-felina	MW432178	MW432178	MW432178
Haberlea rhodopensis	MH757117	MH757117	MH757117
Ligustrum quihoui	MN723864	MN723864	MN723864
Mimulus guttatus	JN098455	JN098455	JN098455
Neobartsia pedicularioides	KP940487	KP940490	KP940492
Olea europaea	MG372117	MG372117	MG372117
Salvia miltiorrhiza	KF177345	KF177345	KF177345
Utricularia reniformis	KY774314	KY774314	KY774314

Sequences newly generated for this study are shown in bold

Table S3. Accession numbers for the nuclear rRNA gene cluster

Species	Accession
**Plantaginaceae**	
Antirrhinum majus	**OK523398**
Aragoa abietina	**OK523399**
Aragoa cleefii	**OK523400**
Callitriche palustris	**OK523401**
Digitalis purpurea	AF193940+KM887394
Hippuris vulgaris	**OK523402**
Linaria vulgaris	**OK523403**
Littorella uniflora	**OK523404**
Littorella uniflora	MT796524
Mecardonia procumbens	**OK523405**
Plantago afra	**OK523406**
Plantago alpina	**OK523407**
Plantago arenaria	**OK523408**
Plantago atrata	**OK523409**
Plantago australis	**OK523410**
Plantago brasiliensis	**OK523411**
Plantago coronopus	**OK523412**
Plantago crassifolia	**OK523413**
Plantago depressa	**OK523414**
Plantago lagopus	**OK523415**
Plantago major	MT937126
Plantago maritima	**OK523416**
Plantago media	**OK523417**
Plantago nubicola	**OK523418**
Plantago ovata	**OK523419**
Plantago rigida	**OK523420**
Plantago rugelii	**OK523421**
Plantago sericea	**OK523422**
Plantago tenuiflora	**OK523423**
Scoparia dulcis	**OK523424**
Veronica chamaedrys	MT796584
Veronica undulata	**OK523425**
Veronicastrum axillare	**OK523426**
**Other Lamiales**	
Dolichandra cynanchoides	MK678751
Eremophila glabra	MN411332
Fraxinus excelsior	MT796551
Melampyrum pratense	MT937124
Myoporum parvifolium	MN411417
Olea europaea	MW646765
Paulownia tomentosa	KP718625
Pogostemon yatabeanus	KP718619
Salvia carduacea	MK257800
Schropularia buergeriana	KP718627
Utricularia minor	MT796583
Verbena stricta	MT610971

Sequences newly generated for this study are shown in bold
